# Primitive neuroectodermal tumor (PNET) of the lung in an adult woman

**DOI:** 10.1186/1477-7819-12-374

**Published:** 2014-12-05

**Authors:** Mariam Gachechiladze, Josef Škarda, Maha Ibrahim, Tomáš Tichý, Patrik Flodr, Pavla Latálová, Jiří Ehrmann, Radek Trojanec, Zdenek Kolář

**Affiliations:** Department of Clinical and Molecular Pathology and Institute of Molecular and Translational Medicine (IMTM), Faculty of Medicine and Dentistry, Palacky University, Hněvotínská 3, 775 15 Olomouc, Czech Republic; School of Cancer Sciences, The University of Birmingham, Edgbaston, B15 2TT Birmingham, UK

**Keywords:** Primitive neuroectodermal tumor, Ewing’s sarcoma, Lung, Differential diagnosis

## Abstract

Primary primitive neuroectodermal tumors (PNETs) are extremely rare in the lung and especially in adult women. We describe a case of PNET of the lung with aggressive behavior in 31-year-old woman. Diagnosis was based on histopathological and immunohistochemical studies, and confirmed by molecular genetic analysis of chromosome rearrangements in the *EWSR1* gene region. Clinical follow-up, post-mortem findings, and differential diagnosis are also discussed.

**Electronic supplementary material:**

The online version of this article (doi:10.1186/1477-7819-12-374) contains supplementary material, which is available to authorized users.

## Background

Primitive neuroectodermal tumors (PNETs) are small-blue-round-cell malignancies, predominantly arising in the soft tissues or bones in children and young adults [1]. PNETs belong to the Ewing’s sarcoma family of tumors, based on shared chromosomal translocation at *EWSR1* (Ewing sarcoma breakpoint region 1). Primary PNETs of the lung are extremely rare, with fewer than 20 cases having been described in English literature to date. Most of these are adolescent or young adult patients, with a male predominance [2–13]. In this case report, we describe a PNET of lung in an adult woman, having a rapid progression of the disease and lethal outcome.

## Case presentation

A 31-year-old woman presented with a 10-month history of right hemithoracic pain and persistent cough, with relative improvement during the day and relative worsening at night. She also reported a weight loss of approximately 11 kg. Computed tomography (CT) and positron emission tomography (PET) imaging of the thorax revealed non-homogeneous opacities and increased accumulation of glucose in the right pleural cavity. Right pleural deposits, found during examination, suggested the presence of viable tumor masses. Directed transparietal biopsy, taken from the glucose accumulation sites seen on the PET scan, was performed.

**Figure 1:**
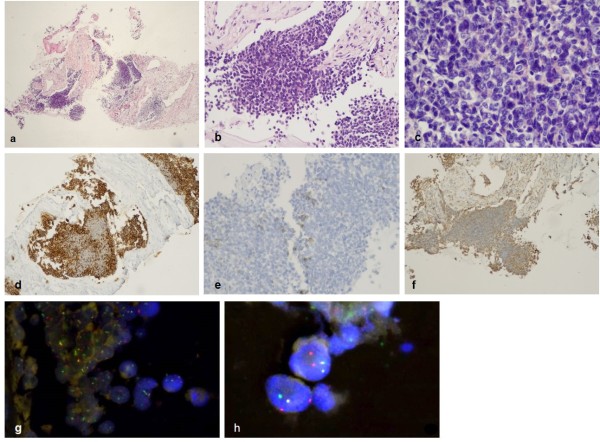
**Biopsy specimen from primitive neuroectodermal tumor (PNET) of the lung. (a)** Areas of highly cellular neoplastic tissue (H&E, magnification × 40), **(b)** discohesive growth of small, blue, round to oval cells, characteristic of pulmonary PNET (H&E, magnification × 200). **(c)** Characteristic scant, basophilic cytoplasm, ovoid or polygonal nuclei, fine granular chromatin texture and marked nucleoli. Some nuclei were hyperchromatic. H&E, magnification × 400. **d)** CD99 membranous expression, characteristic of PNET (magnification × 100). and expression of **(e)** CD56 (magnification × 100) and (**f)** vimentin (magnification × 100). **(g, h)** Fluorescent *in situ* hybridization of *EWSR1* gene: fusion signals: yellow or red/green double spot indicates intact *EWSR1*, separate green and red signals indicate translocation. Magnification **(g) ×** 600, **(h)** × 1000).

Histopathological examination of the biopsy specimens revealed highly cellular neoplastic tissue. The tumor cells were discohesive, showed scant basophilic cytoplasm, and ovoid rather than polygonal nuclei, fine granular chromatin texture, and marked nucleoli. Some of the nuclei were hyperchromatic. There were no evidence of mitotic or apoptotic activity. No obvious signs of necrosis were present, and the stromal tissue adjacent to the tumor was characterized by low cellularity. In some areas, small Homer-Wright rosette-like structures were present. Overall, morphological characteristics were consistent with small-blue-round-cell neoplasm (Figure 1a, b ,c).

A range of immunohistochemical markers were employed for final diagnosis (Table 1), including CD4, CD8, CD10, CD15, CD20, CD34, leukocyte common antigen (CD45), CD56, CD99, cytokeratin 7 (CK7), vimentin, thyroid transcription factor (TTF), synaptophysin, chromogranin, anti-cytokeratin cocktail, High Molecular weight cytokeratin,, calretinin, h-caldesmon, smooth muscle actin (SMA) and mesothelial cell marker (antibody clone: HBME-1). Detailed characteristics and dilutions of antibodies are provided in Additional file 1: Table S1. Immunohistochemical study showed Strong positivity for CD99 (Figure 1d). The neoplastic cells were also focally positive for CD56 and weekly positive for Vimentin (Figure 1e, f). Expression of TTF was extremely weak. All other markers were negative. Proliferative activity, detected by Ki-67 index, was estimated as 5 to 10%. Immunophenotype was characteristic for PNET of the lung.

**Table 1:** **Differential diagnostic markers of small-blue- round-cell tumors in lung**

IHC markers	CD99	CD56	Vim	TTF	Syn	Chr	CD34	LCA (CD45)	CD10	CD15	CK7	AE1/AE3	CK(HMW)	Calret	h-Cal	Desmin	SMA	HBME-1
Tumor types																		
PNET	++	+/-	+	-	+/-	-	-	-	-	-	-	+/-	-	-	-	-	-	-
Desmoplastic small round cell tumor of lung	-	-	++	-	-	-	-	-	-	-	-	++	-	-	-	-	-	-
Small cell carcinoma of the lung	-	+	-	++	+	+	-	-	-	-	-	++	-	+	-	-	-	-
Lung carcinoid	-	-	-	+	++	++	-	-	-	-	-	++	-	-	-	-	-	-
Metastasis of small cell carcinomas of other origin	-	+/-	-	+	-	-	-	-	-	-	+	+	+	-	-	-	-	-
Pulmonary rhabdomyosarcoma	-	-	-	-	-	-	-	-	-	-	-	-	-	-	+	+	-	-
NHL	+/-	-	-	-	-	-	-	++	+	+	-	-	-	-	-	-	-	-

Calret, calretinin; Chr, chromogranin; CK7, cytokeratin 7; CK(HMW), high molecular weight cytokeratin; HBME-1 – mesothelial cell marker; h-Cal, h-caldesmon; IHC, immunohistochemistry; LCA, leukocyte common antigen; NHL, non-Hodgkin’s lymphoma; PNET, primitive neoroectodermal tumor; SMA, smooth muscle actin; Syn, Synaphtophysin; TTF, thyroid transcription factor; Vim, vimentin.

Chromosome rearrangement in the *EWSR1* gene was detected by fluorescent in situ hybridization assay (FISH) to confirm the diagnosis. The results of FISH analysis showed translocation of *EWSR1* in 30% of the tumor cells (Figure 1g, h). More detailed description of molecular analysis in provided in Additional file 1: S2.

Diagnosis was followed by two series of chemotherapy (doxorubicin, cyclophosphamide and vincristine) for 2 days. After 1 month, secondary anemia developed. The patient was treated with hemosubstitution, but her health deteriorated and she died. Post-mortem examination revealed a tumor in the right lung, 210 × 115 × 60 mm in size. The cut surface of the tumor was pinkish colored and areas of hemorrhage were present. Post-mortem and subsequent histopathological examination showed generalized PNET, (Additional file 1: Figure S1), with tumor spread to the mediastinal lymph nodes and subhepatic area. Acute catarrhal purulent bronchopneumonia was present in the left lung. The cause of death was given as cardiorespiratory failure.

## Discussion

PNETs are highly aggressive, usually lethal neoplasms. Although the morphological and immunohistochemical features of pulmonary PNET are not different from its counterparts of other origin, special diagnostic considerations of other tumors with small, blue, round cell morphology and primarily small cell carcinoma of the lung is necessary. Small cell carcinomas usually present at older ages, and usually with a history of smoking, yet they and PNET may exhibit similar histological features. Some PNETs may even show reactivity for cytokeratin and synaptophysin [11, 12]. Hence, a wider range of immunohistochemical markers, including chromogranin and TTF-1 to support a diagnosis of small cell carcinoma, and CD99, vimentin, or FLI1 for PNET should be used for the differential diagnosis. Lung carcinoids share a similar morphology to PNET and form rosettes. However, the detection of chromogranin, synaphtopysin, and neuron specific enolase is necessary to confirm the diagnosis of lung carcinoid. In addition, differential diagnosis is necessary to distinguish PNET from metastasis of small cell carcinomas of other origin. Another important diagnostic consideration for both children and adult patients is pulmonary rhabdomyosarcoma, which is also a rare tumor and belongs to the group of small,-blue,-round-cell tumors. Muscle-specific markers such as desmin, myogenin, or myo-D1, characteristic of rhabdomyosarcoma, should be included in the immunohistochemical study [10]. Lymphoblastic lymphoma and the leukemia group of tumors are also an important diagnostic consideration, as they can be positive for CD99 and negative for epithelial markers. Precursor B-cell lymphoblastic lymphoma is an especially important mimicker of PNET. Like PNET, the malignant cells are small and uniform and have a diffusely infiltrative growth pattern, and they can even form rosette-like structures. Immunohistochemically, precursor B-cell lymphoblastic lymphoma is positive for CD99, and often nonreactive or only focally positive for conventional lymphoma markers such as LCA, CD20, and CD3. Immunopositivity for CD45, CD43, and CD79a are useful for separating lymphoblastic lymphoma/leukemia from PNET [14]. Finally, to confirm the diagnosis of PNET, it is crucial to detect pass break or amplification of *EWSR1* [15]. However, because of the rarity of primary pulmonary PNETs, it is also important to exclude the possibility of metastasis from a bone or soft tissue primary to the lung. Detailed examination by clinical and radiological means should be performed to rule out metastatic tumor from an extrapulmonary primary site.

## Conclusion

Pulmonary PNET should be considered in the differential diagnosis of thoracic tumors, regardless of the age and sex of the patient. Although not specific, CD99 and vimentin are important immunohistochemical markers for diagnosis of PNET. However, a wider range of antibodies should be employed in order to exclude other diagnostic possibilities. Detection of *EWSR1* gene translocation or amplification is the most reliable marker of PNET, including those of pulmonary origin.

## Consent

Oral informed consent was obtained from the responsible person for publication of this Case report and any accompanying images.

## Electronic supplementary material

Additional file 1: **Supplementary materials.** (PDF 256 KB)
