# Supplementary material for: Primitive neuroectodermal tumor (PNET) of the lung in an adult woman
Source: World J Surg Oncol. 2014 Dec 5;12:374. doi: 10.1186/1477-7819-12-374 (PMC4289161; doi:10.1186/1477-7819-12-374)
Supplement: Supplementary file 1 — Additional file 1: Supplementary materials. (PDF 256 KB) [file 12957_2014_1850_MOESM1_ESM.pdf]

Supplementary materials

Table 1. Antibodies used for immunohistochemical diagnosis of PNET.

**Antgen**	**Antibody Clone**	**Dilution**	**Manufacturer**
CD99	12E7	1:50	®Dako
CD56	1B6	1:50	®NovoCastra
Vimentin	V9	1:10	®Dako
TTF	8G7G3/1	prediluted	®Covance
synaptophysin	SY38	1:40	®Dako
chromogranin	5H7	1:50	®NovoCastra
CD34	56C6	1:50	®Dako
leucocyte common antigen (LCA)	CD45	1:100	®Dako
CD10	L26	1:100	®Dako
CD15	BRA4F1	1:100	®BioGenex
CK7	OV-TL12/30	1:100	®Dako
anti-cytokeratin cocktail	AE1/AE3	1:100	®BioGenex
CKV	33bE12	1:100	®Dako
calretinin	5A5	1:100	®NovoCastra
h-caldesmon	h-CD	1:100	®Dako
SMA	1A4	1:50	®Dako
Mesothelial cell	HBME-1	1:100	®Dako

Molecular analysis

Chromosome rearrangements in the EWSR1 gene region (Ewing sarcoma breakpoint region 1) were detected by fluorescent in situ hybridization assay (FISH). LSI EWSR1 Break Apart FISH Probe Kit (VYSIS, Abbott Molecular) was used. The analysis was performed on formalin-fixed, paraffin-embedded (FFPE) tissue. The tissue section was immobilized on positively charged slide, rehydrated,

digested by using HCl and pepsine and hybridized with the LSI EWSR1 probe. The signals were detected in fluorescence microscope. 100 non-overlapping nuclei were evaluated in the sample. According the probe manufacturer, fusion signal was considered as intact EWSR1 gene and two split signals (orange and green) indicated a rearrangement of the EWSR1 region. Positive interpretation was defined as >15% nuclei with split signals (*Bridge et al., 2006*).

Autopsy Findings

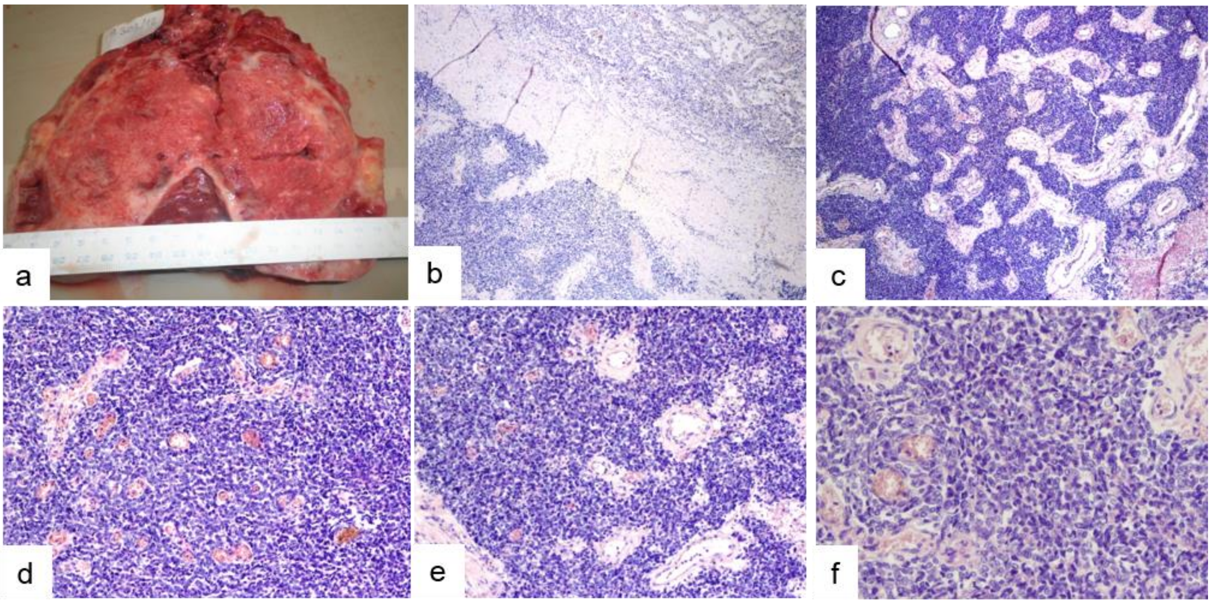

Autopsy Findings. **a.** Gross appearance, tumor deposits involve whole right lung, **b.** highly cellular neoplastic tissue, H&E, 40x, **c.d.** tumor is composed with small, blue round to oval cells, prominent vascularisation is also present, H&E, 100x, **e.f.** dyscohesive tumor cells, and small Homer-Wright like rossetes, H&E, x200,
